# Epidemiology of Kidney Cancer

**DOI:** 10.1155/2008/782381

**Published:** 2008-11-04

**Authors:** D. Pascual, A. Borque

**Affiliations:** ^1^Department of Urology, San Pedro Hospital, 26006 Logroño, Spain; ^2^Department of Urology, Miguel Servet University Hospital, 50009 Zaragoza, Spain

## Abstract

Some tumors are known to have a definite cause-effect etiology, but renal cell carcinoma (RCC) is not one of them precisely. With regard to RCC we can only try to identify some clinical and occupational factors as well as substances related to tumorigenesis. Smoking, chemical carcinogens like asbestos or organic solvents are some of these factors that increase the risk of the RCC. Viral infections and radiation therapy have also been described as risk factors. Some drugs can increase the incidence of RCC as well as other neoplasms. Of course, genetics plays an outstanding role in the development of some cases of kidney cancer. Chronic renal failure, hypertension, and dialysis need to be considered as special situations. Diet, obesity, lifestyle, and habits can also increase the risk of RCC. The aim of this review is to summarize the well-defined causes of renal cell carcinoma.

## 1. INTRODUCTION

Speaking about cancer, one of the most difficult issues is to find a definite and direct cause. There are few tumors with a well-known etiology, but renal cell carcinoma (RCC) is not one of them precisely.

In these cases, we can only try to identify some clinical and occupational factors, or some substances related to carcinogenesis.

Epidemiology is an important tool to answer many questions about cancer origin. Differences in age, gender, and geographic distribution have been reported, and multiple clinical factors related to the development of RCC have been established. Some of them have been thoroughly demonstrated in experimental models and in vitro studies, however not all of them recognized as definite etiologic factors.

## 2. MATERIAL AND METHODS

A systematic review search strategy was developed to identify publications related to epidemiology of renal cell carcinoma. This search strategy was run in PubMed through the medical subject heading “carcinoma, renal cell” and the subheading of this descriptor “epidemiology.” We limited our search strategy to articles published in the previous 5 years, language English or Spanish, and related to humans.

585 articles were found. Abstracts were evaluated and the full text of articles selected was reviewed. Secondary search from the bibliography of selected articles was also considered.

The European cancer registry-based study on survival and care of cancer patients (EUROCARE) and our experience was considered. Last review was on 31 of March 2008.

## 3. DEMOGRAPHIC ASPECTS OF RENAL CELL CARCINOMA

Among urologic tumors, RCC takes the third place in incidence, following prostate carcinoma and transitional cell carcinoma of bladder.

Representing two percent of the adult malignancies [1], this malignancy takes the tenth and fourteenth place among men and women, respectively, with a man to woman ratio of 3/2 [2]; see Table 1.

The peak incidence occurs in the sixth decade, with 80% of the cases within the 40 to 69-year-old population.

Although the most frequent renal tumor in the childhood is the Wilms tumor, it is important to state that the RCC represents between 2% to 6% of the renal tumors in children, without differences between sexes [3, 4]. Besides, the incidence of both malignancies is similar in the second decade of life. In these early ages the papillary differentiation seems to be more frequent with higher tendency to present a locally advanced and high-degree disease at the moment of the diagnosis [5], However, when comparing stage by stage with adult tumors, we find a better response to surgical treatment and higher survival rates, even with positive nodal disease.

RCC represents 85 to 90% of renal parenchymal malignancies [6, 7].

Among urologic tumors, it is the worst in cancer specific mortality, since more than 40% of the patients with RCC die of the disease, opposite to the 20% mortality observed in prostate cancer or bladder carcinoma.

In United States 30 000 new cases are diagnosed every year, and approximately 12 000 patients die of this disease, with an incidence of near nine cases per 100 000 inhabitants per year. Afroamericans have 10 to 20% higher incidence, and the reason is not completely understood [9].

Most of the cases of RCC are sporadic and only 4% are familiar. The estimated number of new cases in the European Union during 2006 was 63300, with 26400 deaths of RCC [10]. The estimated survival in 5 years rises to 54% in males and 57% in women [11].

Table 2 shows different incidences of RCC in the world.

Since 1930 incidence of RCC has been increasing, mostly between 1930 and 1980. Within this period the incidence also rose from 0.7 to 4.2 per 100 000 per year in women and from 1.6 to 9.6 in men [12]. Since 1980 a sharp increase has not been observed comparing to other genitourinary tumors or other type of malignancies. Similarly, deaths caused by RCC had been stable.

Variations of incidence within the first period could be explained by an easier diagnosis, as a result of diffusion and routine use of diagnostic tools such as ultrasound or CT scan, and not due to a real increased incidence of RCC.

It is also important to state that RCC is found incidentally in 1.5% of the autopsies [13].

RCC is more frequent in urban populations rather than in rural ones. This observation may be explained by the sanitary conditions and the smoking habit in urban populations. However it has not been related neither to socioeconomic nor to educational status [14].

There are multiple factors related to the development of RCC; see Table 3. Some of them have been demonstrated in experimental models and in vitro studies, however not all of them can be considered as definite etiologic factors.

Herein, we describe these main factors.

### 3.1. Smoking

Multiple carcinogenic substances have been identified in tobacco and related to a variety of neoplasms at different levels. A high incidence of RCC in smokers has been shown [16], estimated in 2.3 fold risk ratio, directly related with the number of cigarettes and inversely with age of beginning of the habit. Likewise it has been shown that the carcinogen dimetilnitrosamine induces this neoplasm in experimental studies. Some authors reported that smokers' risk for RCC compares to nonsmokers' after the fifth year of nonsmoking, but a meta-analysis made by Hunt showed that only after ten years the risk can be similar in both groups [17], depending on the dose of tobacco inhaled. Another study by McLaughin [18] and Lipworth [19] confirmed tobacco as the most important risk factor for renal cancer, detected in 20% of the cases of RCC.

But smoking is not only important in the genesis of RCC, and prognostic nomograms have also been developed [20]. A multivariate study carried out in “Miguel Servet” University Hospital of Zaragoza, Spain (in press), smoking habit increases 2.84 fold (1.27–6,32) the risk of progression of the disease after surgery [21], similarly to previous studies in other countries.

### 3.2. Chemical carcinogens

Some radiological contrasts have been associated with an increased incidence of RCC [22]. Although Cycasin (a substance derived from a palm fruit that grows in the island of Guam) induces RCC in animals, a higher incidence of this neoplasm within the island population could not be shown.

Cadmium was demonstrated to have influence on the development of RCC in smokers [23, 24].

- *Asbestos*. A significantly elevated mortality rate for kidney cancer has been reported in two cohort studies, on insulation workers [25] and on asbestos products workers [26]. Autopsy surveys and animal studies indicate that asbestos fibers can be deposited in kidney tissue.
- *Organic solvents*. Pesticides, copper sulphate, benzidine, benzene herbicides, and vinyl chloride have been found as risk factors of RCC in prolonged exposure. A dose dependent effect has been seen only for organic solvents and copper sulphate [27, 28].
Recent reviews of cohort studies found little or no evidence of an increased risk for RCC among people exposed to gasoline and petroleum derived products [29, 30].
- *Polycyclic aromatic hydrocarbons*. Workers exposed to high levels of polycyclic aromatic hydrocarbons like coke and coal oven workers, firefighters, asphalt, and tar have been reported to be at increased risk for kidney cancer.

### 3.3. Radiation

Ionizing radiation appears to increase the RCC risk slightly, especially among patients treated for ankylosing spondylitis and cervical cancer [31]. An increased risk has also been reported for patients receiving radium 224 for bone tuberculosis and ankylosing spondylitis [32].

### 3.4. Viruses

The immunosuppressant state related to the HIV infection determines that prevalence of RCC in the infected population rises 8.5 times compared to the prevalence of the noninfected ones.

The influence of the polyomavirus SV 40 and of the adenovirus 7 has also been detected in experimental studies.

A clear-cut association was found between herpes-type virus and renal tumors in toads. These findings led to search for evidence of herpes virus proteins in human tumors as well. Although herpes simplex proteins were found in only one study [33, 34], these findings need to be confirmed by further research.

### 3.5. Diuretics

This type of drugs which inhibits water reabsorption on the renal tubule cells seemed to be responsible for a higher incidence of RCC in patients with chronic intake of diuretics [35, 36]. Even though, it is noteworthy that hydrochlorothiazide and furosemide (both effective at the renal tubule level) induce tubular cell adenomas and adenocarcinomas of the kidney in rats [37]. But Yuan [38] showed in his study that an adequate use of diuretics for treating hypertension eliminates the risk associated with the above mentioned drugs, differentiating the influence of hypertension as a risk factor for RCC rather than diuretics.

### 3.6. Analgesics

This is a controverted topic. Several studies reported an increased incidence of RCC in patients with chronic intake of analgesics like paracetamol, salicylates, or phenacetin [39, 40], however in other studies this relationship has not been confirmed neither for time of consumption nor for dose of the drug taken [41].

Although a heavy use of drugs containing phenacetin has been clearly demonstrated to increase the risk for transitional cancer of the renal pelvis, the association with RCC is much weaker. On the other hand, an increased risk of RCC associated with aspirin or acetaminophen consumers was observed [42], but others believe that neither acetaminophen nor other analgesics have been convincingly linked with RCC [19].

### 3.7. Oestrogens (dietilestilbestrol)

Although oestrogens can induce RCC in the animal model, little evidence supports an association of the disease with oestrogens in humans [43] and only weak relation has been reported for the use of oestrogens after menopause and for oral contraceptives [44].

### 3.8. Inheritance

Most of the cases of RCC are sporadic; however there are some defined types of RCC with a hereditary pattern [45].

**(1) Von Hippel-Lindau (VHL) disease**

The VHL disease is inherited through an autosomal dominant trait. The syndrome is caused by germline mutations of the VHL tumor suppressor gene, located on chromosome 3p25-26; these mutations can virtually always be identified [46]. The VHL protein takes part in cell cycle regulation and angiogenesis [47]. Patients develop capillary haemangioblastomas of the central nervous system and retina, clear cell carcinoma, phaeochromocytoma, pancreatic, and inner ear tumors.

The clinical diagnostic criteria of VHL disease consist of

- presence of capillary haemangioblastoma in the central nervous system or retina,
- presence of one of the typical VHL associated extraneural tumors, within pertinent family history.

Fourty to sixty percent of the patients with VHL disease present an RCC. Although they are usually low-grade tumors, the progress rate to metastasis is around 30% [48].

Renal lesions in carriers of VHL germline mutations are either cysts or clear cell RCC. They are typically multifocal and bilateral.

**(2) Hereditary papillary renal carcinoma**

This type of renal carcinoma is an inherited tumor syndrome with autosomal dominant trait and of late onset, with multiple and bilateral papillary renal cell carcinomas type 1. The disease is caused by activating mutations of the MET oncogene which maps the chromosome 7q31.

**(3) Hereditary leiomyomatosis and renal cell carcinoma**

This is an autosomal dominant tumor syndrome with germline mutations in the FH gene (chromosome lq42.3–q43), These patients have the tendency to acquire benign leiomyomas of the skin and the uterus, and occasionally papillary renal cell carcinoma type 2 and uterine leiomyosarcomas.

**(4) Birt-Hogg-Dube syndrome**

This syndrome is characterized by benign skin tumors, specifically fibrofolliculomas, trichodiscomas, and acrochordons. Multiple renal tumors and spontaneous pneumothoraces are also frequent. We can find chromophobe RCC, typical RCC, hybrid oncocytoma, papillary RCC, or oncocytic tumors.

The Birt-Hogg-Dube gene maps the chromosome 17p11.2 and encodes the protein called folliculin. This gene is also involved in sporadic RCC [49].

**(5) Familiar clear cell renal cell carcinoma**

These families present a hereditary form of multiple, bilateral clear cell RCC but without any clinical evidence of suffering the von Hippel-Lindau disease.

This hereditary cancer is characterized to present translocations affecting the chromosome 3. Translocations have been described among the chromosome 3 and the chromosomes 8 [50], 6 [51, 52], 2 [53, 54], 1 [55], and 4 [48].

### 3.9. Acquired cystic disease/chronic dialysis

Approximately the 35 to 47% of the patients on dialysis and specially those with a very long history present acquired cystic disease. Some patients with this disease develop a papillary hyperplasia in the epithelium of the cysts that would be the origin of the RCC [56, 57].

Approximately the 5 to 9% of the patients with acquired cystic disease will develop an RCC [58], showing a higher incidence than the general population. As such, we suggest a close follow-up in the kidney-transplant population, and therefore the immune-suppressed individuals who are on dialysis for a long time, due to a high risk of developing RCC.

### 3.10. Diet and obesity

Hypercaloric diet and obesity seem to be associated with a higher risk for suffering of RCC. Obesity accounts for about 30% of renal cancers [19].

Some studies relate a higher incidence of RCC with high body mass index. The relative risk was found to be 3.3 in males and 2.3 in females [59].

The mechanism of obesity to cause kidney cancer is not clear. Hormonal changes such as increased levels of endogenous oestrogens in the obese may be the mechanism through which oestrogens induce renal cancer as observed in animal models. However, there is scant epidemiologic evidence supporting hormonal carcinogenesis regarding RCC. Obesity may also predispose to arterionephrosclerosis, which may render the renal tubules more susceptible to carcinogens. Elevated cholesterol levels associated with obesity might also play a role, as suggested by animal studies showing that cholesterol-lowering drugs provide some protection against RCC. Cholesterol and other lipids may favour tumor development by an inhibitory effect on immune cells.

Low vitamin D level, which is usually present in obese patients, may predispose to acquire RCC. This vitamin is known to have inhibitory effects on the growth of RCC cell lines in vitro [60].

Finally, Lipworth [19] reported that the only consistent protective factor is consumption of fruit and vegetables.

### 3.11. Coffee, alcohol, and other beverages

Case-control studies have not confirmed the suggested relationship between kidney cancer and consumption of coffee, when adjusting the smoking variable. Although two studies have suggested a positive association, a two-fold increased risk in both sexes was associated with the use of decaffeinated coffee. In another study, an increased risk of RCC was found only among women with regular coffee intake [61]. No dose-dependent risk was reported in either study.

On the other hand, a significant lower risk was reported in Norway among consumers of seven or more cups of coffee compared to those who drink two or fewer cups daily, representing a relative risk of 0.25 [62]. Review studies indicate that coffee consumption does not increase RCC risk.

Few studies have shown an increased risk of RCC among tea women consumers [63]. Another study has found a dose-response relationship between tea consumption and kidney cancer mortality [64]. The etiologic significance of these findings in direct relationship with tea tannins is not clear [65].

The association between alcoholism and kidney cancer mortality has not been demonstrated by well-deisgned studies [66]. In fact, a recent case-control study found a statistically significant inverse association between alcohol consumption and RCC risk [67]. No increase in mortality from kidney cancer has been reported either within alcoholic patients or brewery workers [68].

### 3.12. Physical activity

A moderate recreational activity reduces the risk of renal cancer both in men and women. The mechanism is not clear, but there is no doubt that energy expenditure is one of the major determinants for obesity, which is a strong risk factor for RCC.

### 3.13. Hypertension

Hypertension seems to be significant for the development of kidney cancer. The strength of this relationship is reduced with the use of diuretics and other antihypertensive drugs, regardless of some of these drugs have been associated with RCC risk. The main problem consists to identify whether the increased risk is due to hypertension or antihypertensive medications.

Despite the mechanism for hypertension to cause renal cancer is not completely understood [69] it seems that metabolic and/or functional changes in the renal tubular cells produce carcinogenesis. Wide case-control studies have only found a slight relation between RCC and hypertension [70]. Further studies are needed.

### 3.14. Alterations in development of the kidney

The anomalous development of the kidneys may act as a teratogenic factor.

In horseshoe kidneys, the area of the isthmus is prone to develop tumors [71], due to an anomalous migration of the cells toward this area. However, although the most frequent tumor developed in this malformation is the RCC, the incidence remains identical to that of the general population, without differences in evolution or prognosis [72].

In conclusion we can affirm that respect RCC, like in other malignant diseases, ethiology, and risk factors are not completely understood. There is some evidence that certain situations, drugs, habits, or genetics are related to the development of renal cancer, but several studies found controversial results and different degrees of evidence.

Smoking and obesity seem to be the most important independent risk factors in the genesis of RCC, reported by different authors.

Chromosomal mutations were clearly identified in the context of well-defined hereditary diseases.

The adequate use of diuretics and analgesics may be recognized as protective factors not only for RCC but for other diseases as well.

General healthy habits like limiting alcohol and coffee intake, decreasing the number of cigarettes, lowering fat consumption, keeping a suitable weight, and practicing regular exercise may reduce the risk and indicence of RCC.

## Figures and Tables

**Table 1:** Epidemiologic features of the RCC.

3rd urologic tumor in incidence	Maximum incidence on 6th decade
2% of adults malignancies	Male/Female ratio: 3/2
85–90% of adult renal parenchymal malignancies	10th male malignancy and 14th female malignancy
2–6% of child renal malignancies	More frequent in afroamerican people
Cancer specific mortality of 40%	More frequent in urban people

**Table 2:** Geographic distribution of RCC [8].

Incidence	Zones
High	Denmark, New Zealand, Norway, Scotland
Moderate	United States, Australia, Belgium, France, Holland
Low	Spain, Ireland, Italy, Japan, Venezuela, India, China

**Table 3:** Some etiologic factors of RCC [15].

Etiologic factor	Relative risk (C.I. 95%)
Von Hipple-Lindau disease	100 (not available)
Chronic dialysis	32 (not available)
Obesity	3.6 (2.3–5.7)
Smoking	2.3 (1.1–5.1)
First relative with RCC	1.6 (1.1–2.4)
Hypertension	1.4 (1.2–1.7)
Dry cleaners	1.4 (1.1–1.7)
Diuretics	1.3 (1.07–1.52)
Trichloroethylene	1 (0.7–9.66)
Radiation therapy	1.1 (3.2–8.1)
Phenacetin	1.1 (2.6–6)
Polycystic kidneys	0.8 (2–4.5)
Cadmium exposure	1 (2–3.9)
Arsenic exposure	1.6 (2.3–4.1)
Asbestos exposure	1.1 (1.4–1.8)
